# The peculiar challenge of bringing CAR-T cells into the brain: Perspectives in the clinical application to the treatment of pediatric central nervous system tumors

**DOI:** 10.3389/fimmu.2023.1142597

**Published:** 2023-03-21

**Authors:** Giada Del Baldo, Francesca Del Bufalo, Claudia Pinacchio, Andrea Carai, Concetta Quintarelli, Biagio De Angelis, Pietro Merli, Antonella Cacchione, Franco Locatelli, Angela Mastronuzzi

**Affiliations:** ^1^ Department of Pediatric Haematology and Oncology, and Cell and Gene Therapy Bambino Gesù Children’s Hospital, Scientific Institute for Reasearch, Hospitalization and Healthcare (IRCCS), Rome, Italy; ^2^ Department of Experimental Medicine, Sapienza University of Rome, Rome, Italy; ^3^ Department of Neurosciences, Neurosurgery Unit, Bambino Gesù Children’s Hospital, Scientific Institute for Reasearch, Hospitalization and Healthcare (IRCCS), Rome, Italy; ^4^ Department of Life Sciences and Public Health, Catholic University of the Sacred Heart, Rome, Italy

**Keywords:** pediatric brain tumors, CAR-T cells, blood-brain barrier, glymphatic system, neurotoxicity

## Abstract

Childhood malignant brain tumors remain a significant cause of death in the pediatric population, despite the use of aggressive multimodal treatments. New therapeutic approaches are urgently needed for these patients in order to improve prognosis, while reducing side effects and long-term sequelae of the treatment. Immunotherapy is an attractive option and, in particular, the use of gene-modified T cells expressing a chimeric antigen receptor (CAR-T cells) represents a promising approach. Major hurdles in the clinical application of this approach in neuro-oncology, however, exist. The peculiar location of brain tumors leads to both a difficulty of access to the tumor mass, shielded by the blood-brain barrier (BBB), and to an increased risk of potentially life-threatening neurotoxicity, due to the primary location of the disease in the CNS and the low intracranial volume reserve. There are no unequivocal data on the best way of CAR-T cell administration. Multiple trials exploring the use of CD19 CAR-T cells for hematologic malignancies proved that genetically engineered T cells can cross the BBB, suggesting that systemically administered CAR-T cell can be used in the neuro-oncology setting. Intrathecal and intra-tumoral delivery can be easily managed with local implantable devices, suitable also for a more precise neuro-monitoring. The identification of specific approaches of neuro-monitoring is of utmost importance in these patients. In the present review, we highlight the most relevant potential challenges associated with the application of CAR-T cell therapy in pediatric brain cancers, focusing on the evaluation of the best route of delivery, the peculiar risk of neurotoxicity and the related neuro-monitoring.

## 1 Introduction

Brain tumors are the most common solid tumors in childhood (1). Treatment and prognosis depend on the histological type and molecular profile of the neoplasm, ranging from a watch-and-wait approach after initial surgery, for low-grade tumors, to toxic therapeutic approaches including chemotherapy and radiotherapy in high-grade tumors. Despite aggressive multimodal treatment, high-grade central nervous system (CNS) tumors remain the leading cause of childhood cancer-related death (2). Many subtypes, such as high-grade gliomas (HGG) and diffuse intrinsic pontine gliomas (DIPGs), continue to have a dismal prognosis, with 5-year overall survival <20% and 5%, respectively (3). These tumors are characterized by a strong intrinsic genetic and phenotypic heterogeneity, recently proved to be involved in the enhanced tumorigenicity and resistance to therapy typical of these tumors (4), explaining the difficulty of eradicating the disease.

Furthermore, the intensive treatment approaches conventionally in use, in particular the brain irradiation, are associated with devastating long-term morbidities, including endocrine, psychiatric, cognitive and developmental disorders and neurological impairment, as well as secondary tumors (5). Overall, these findings underscore the still unmet need to identify innovative treatment strategies to improve the outcome of children affected by high-grade CNS tumors, sparing them from the burden of long-term sequelae.

Immunotherapies, exploiting the capacity of immune system to attack cancer cells, have become the focus of a wide stream of translational research. The advances in cancer immunotherapy have improved outcomes for several human cancers, and in some cases have produced dramatic responses in patients highly refractory to all conventional treatments (6, 7). The most remarkable success of immunotherapies resulted in significant improvement of overall survival in phase II–III trials in some tumors, such as melanoma (7) and leukemia (8).

In adult cancers, high mutational burden and expression of immune checkpoint molecules have been shown to correlate with response to certain immunotherapy approaches, especially checkpoint inhibitors (9). Some of the most clinically aggressive pediatric brain tumors, including DIPG, HGG and medulloblastoma (MBL), do not show a highly immunosuppressive nor inflammatory immune tumor microenvironment (TME), representing immunologically ‘cold’ tumors (10, 11). Moreover, the TME tends to vary among the different types of pediatric brain tumors (12). In recent years, our knowledge of molecular patterns of pediatric CNS tumors has increased, as result of high-resolution genomic, epigenetic and transcriptomic profiling, allowing a more specific classification of these tumors. Interestingly, large-scale sequencing studies of pediatric tumors identified novel driver genetic mutations, but underscored that pediatric cancers, including brain tumors, typically have very few somatic mutations (13, 14). This low mutational burden is thought to represent the reason for the lower immunogenicity demonstrated by pediatric cancers, as compared to adult cancers (15), and for the unsatisfactory results obtained with checkpoint inhibitors (16).

Discouraging results observed with immune checkpoint inhibition in high-grade tumors have oriented interest on other types of immunotherapy and several evidences encourage the development of new strategies for treatment of brain tumors, ranging from monoclonal antibodies (mAb) to cellular therapy.

Chimeric antigen receptor T cell (CAR-T) therapy provided unprecedented results in relapsed/refractory B-cell acute leukemia and high-grade B-cell non-Hodgkin lymphoma (NHL) (17). These outstanding results opened up the interest of CAR-T cell therapy also in solid tumors, but, unfortunately, not with comparable results (18). Major limitations of CAR-T cells in solid cancer include: i) the heterogeneous target antigen expression, ii) the difficult trafficking and penetration of CAR-T cells into the tumor, iii) the low CAR-T cell expansion and persistence due to immunosuppressive and hypoxic tumor microenvironment (19, 20). All these conditions are even more pronounced in brain tumors (21). In addition, several specific challenges exist in targeting CNS tumors with immunotherapy, such as the peculiarity of the brain location, the presence of the blood-brain barrier (BBB) and the risks associated with the occurrence of an inflammatory reaction in the CNS. All these aspects will be outlined in the present review.

## 2 Chimeric antigen receptors

**Figure 1:**
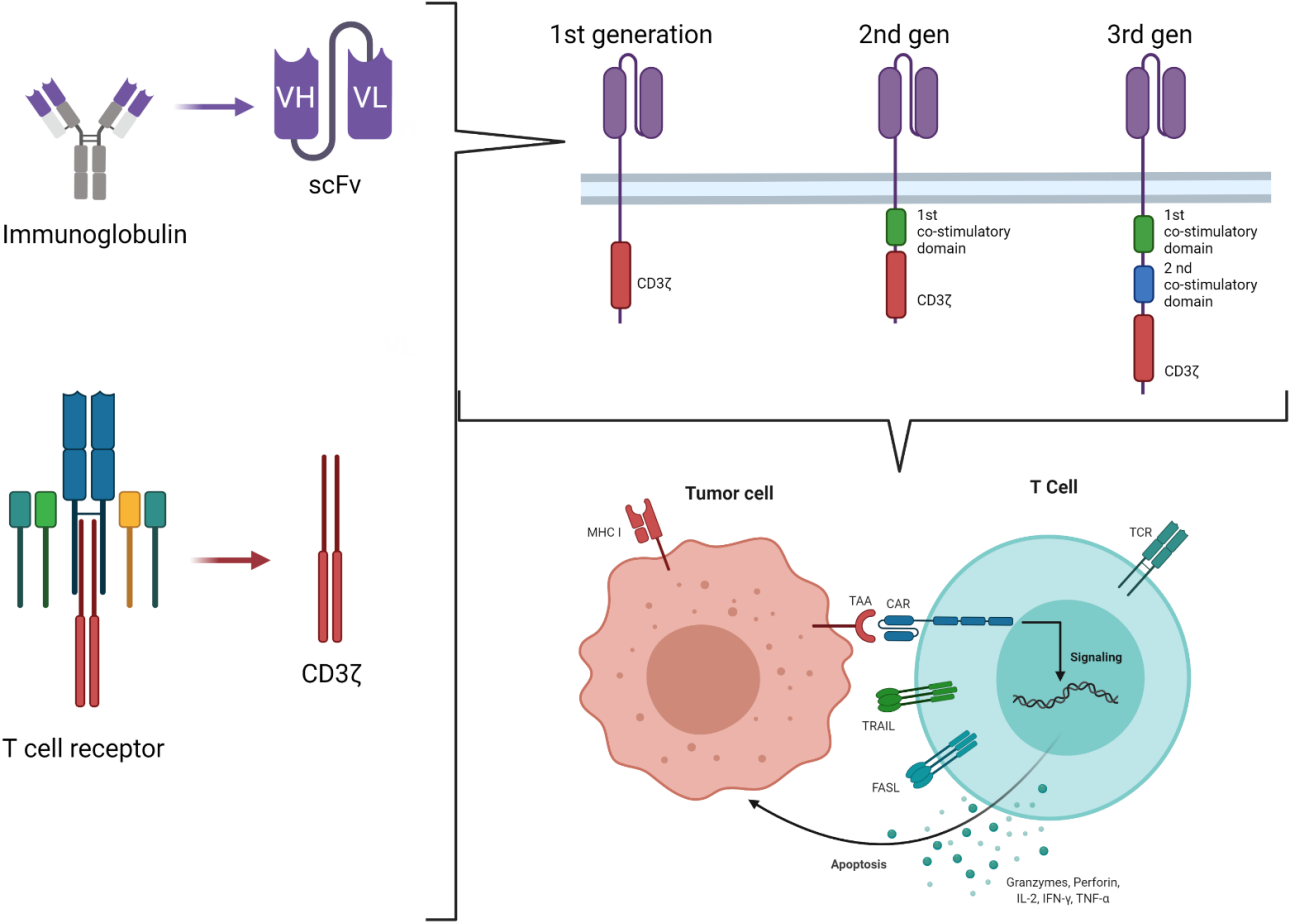
Second and third generation CAR-T cell construct.

Chimeric antigen receptors derive by the combination of the Major Histocompatibility Complex (MHC)-independent antigen recognition due to the ScFV portion of mAb and the effector function of T cells, originally ideated by Eshhar et al. in 1989 (22). It is based on the engineering of T cells to express a chimeric molecule resulting from the fusion of the heavy and light chain variable regions of a mAb to the cytotoxic zeta chain (ζ) of a T cell receptor (TCR) (first generation CAR) (**Figure 1**) (23). Using this strategy, it is possible to target a wide range of tumor-associated targets expressed on the surface of tumor cells and to potentially overcome the low immunogenicity of tumors related to the low levels of antigen and their poor presentation by MHC molecules. The advantages of CAR-T cells over mAb are multiple and rely on the ability of CAR-T cells to self-amplify upon activation and to better distribute in the tissues. Moreover, cytokines secretion upon T-cell activation associated with the tumor antigen encounter recruits additional components of the immune system, improving the anti-tumor response. Lastly, CAR-T cells may enter the memory pool and provide long lasting protection against tumor re-growth.

Engagement of the CAR by its ligand on the tumor cell results in tyrosine phosphorylation of immune-receptor activation motifs present in the cytoplasmic domain, initiating T-cell signaling and specific tumor cell lysis *via* the perforin/granzyme pathways. However, tumors rarely express costimulatory molecules. The presence of the signal 1 (ζ chain of the TCR) in the absence of the signal 2 (costimulatory molecules signaling) enables CAR-T cells to be activated and kill tumor cells, but not to proliferate and expand. This observation led to the development of second-generation CARs incorporating signaling domains of the T-cell costimulatory molecules in tandem with the CD3 ζ chain, so that, upon ligation, T cells both kill and proliferate (24). Intracytoplasmic signaling domains of CD28, CD134 (OX40), CD137 (4-1BB), inducible costimulatory (ICOS), CD27, DAP10 or CD244 (2B4), in various combinations, have been used (25, 26). However, it is well known that, in the setting of solid tumors, second generation CARs do not always expand properly after infusion into patients, and it is well accepted that antitumor efficacy requires adequate expansion and persistence *in vivo* (27). Understanding how the CAR structure influences these properties is therefore key for the future designs of CAR-T cells. An example is represented by the evidence of the primary role of T-cell exhaustion in limiting the antitumor efficacy of T cells in the setting of chronic antigen exposure (28, 29). Recently, the central role of the CAR structure in predisposing chronic T-cell activation and exhaustion has been demonstrated, proving that CD28 co-stimulation augments, whereas 4-1BB co-stimulation reduces, exhaustion induced by persistent CAR signaling (30). In the search for the optimal design for a CAR, even more potent constructs were developed, containing two costimulatory domains (**Figure 1**). These so-called third generation CAR constructs provide more potent proliferation in response to tumors than either first or second generations; moreover, higher T-cell resistance to several tumor evasion strategies was observed (31). However, while the superiority of second-generation CAR over the first has been clearly proved, whether the incorporation of additional costimulatory domains in the third generation provides further benefits remains to be definitely documented (27). The optimal design of a given CAR remains an area of active investigation and should be empirically evaluated for the treatment of different malignancies (32).

## 3 CAR-T cells antigens for CNS tumors

In contrast to hematological diseases, brain tumors are characterized by antigenic heterogeneity on the cell surface, with B7-H3 and GD2 being the most consistently expressed antigens (33).

CAR-T cells were designed for different type of antigens, all of these tested in pre-clinical and/or clinical trials: epidermal growth factor receptor variant III (EGFRvIII), human epidermal growth factor receptor 2 (HER2), interleukin 13 receptor alpha 2 subunit (IL13R 2), EGFR806, B7-H3 and disganglioside-GD2.

In a recent study published by Haydar et al. the mean antigen expression on tumor cells obtained from pediatric brain tumor patient-derived orthotopic xenograft models was 68% for B7-H3, 74.1% for GD2, 37.5% for IL-13Rα2, 50.1% for EphA2 and 36.1% for HER2 (33).

Recently, new potential targets, such as CXCL5/CXCL6 and PTK7, were explored (34, 35). The heterogenous antigen expression prompted the use of simultaneous multiple targeting that demonstrate increase antitumor potency, reducing the possibility of tumor escape in pre-clinical mouse models (36–39).

CAR-T cells showed *in vitro* anti-tumor activity against glioblastoma and medulloblastoma by targeting HER2, EGFR806 and B7-H3. Phase I clinical trials are now ongoing to study the efficacy of these antigen-specific CAR-T cells in children and young adults with recurrent or refractory CNS tumors.

An overview of published and ongoing phase 1 clinical trial on CAR-T cell for pediatric brain is detailed in **Table 1**.

**Table 1:** Overview on CAR-T cells clinical trial for pediatric brain tumors.

Trial	Center	Target	Route of delivery	Tumor type	Recruitment status
NCT04185038	Seattle Children’s Hospital	B7H3	Locoregional	Diffuse intrinsic pontine glioma/diffuse midline glioma and recurrent or refractory pediatric CNS tumors	Recruiting
NCT03500991	Seattle Children’s Hospital	HER2	Locoregional	HER2-positive recurrent/refractory pediatric CNS tumors	Recruiting
NCT03638167	Seattle Children’s Hospital	EGFR806	Locoregional	EGFR-positive recurrent or refractory pediatric CNS tumors	Recruiting
NCT04510051	City of Hope Medical Center	IL13Rα2	Intraventricularly	Recurrent or refractory malignant glioma	Recruiting
NCT04099797	Baylor College of Medicine	GD2	Intravenously	GD2-positive brain tumors	Recruiting
NCT02442297	Baylor College of Medicine	HER 2	Locoregional	HER2-positive brain tumors	Recruiting
NCT04196413	Stanford University	GD2	Intravenously or intracerebroventricularly	DIPG and DMG	Recruiting
NCT01109095	Baylor College of Medicine	HER2	Intravenously	Recurrent or progressive HER 2 positive glioblastoma	Closed
NCT02208362	City of Hope Medical Center	IL13Rα2	Locoregional	Recurrent/refractory high-grade glioma	Closed
NCT04903080	Pediatric Brain Tumor Consortium	HER2	Intravenously	Ependymoma	Recruiting

## 4 Trafficking through BBB and glymphatic system: Has the brain still to be considered an immune sanctuary?

**Figure 2:**
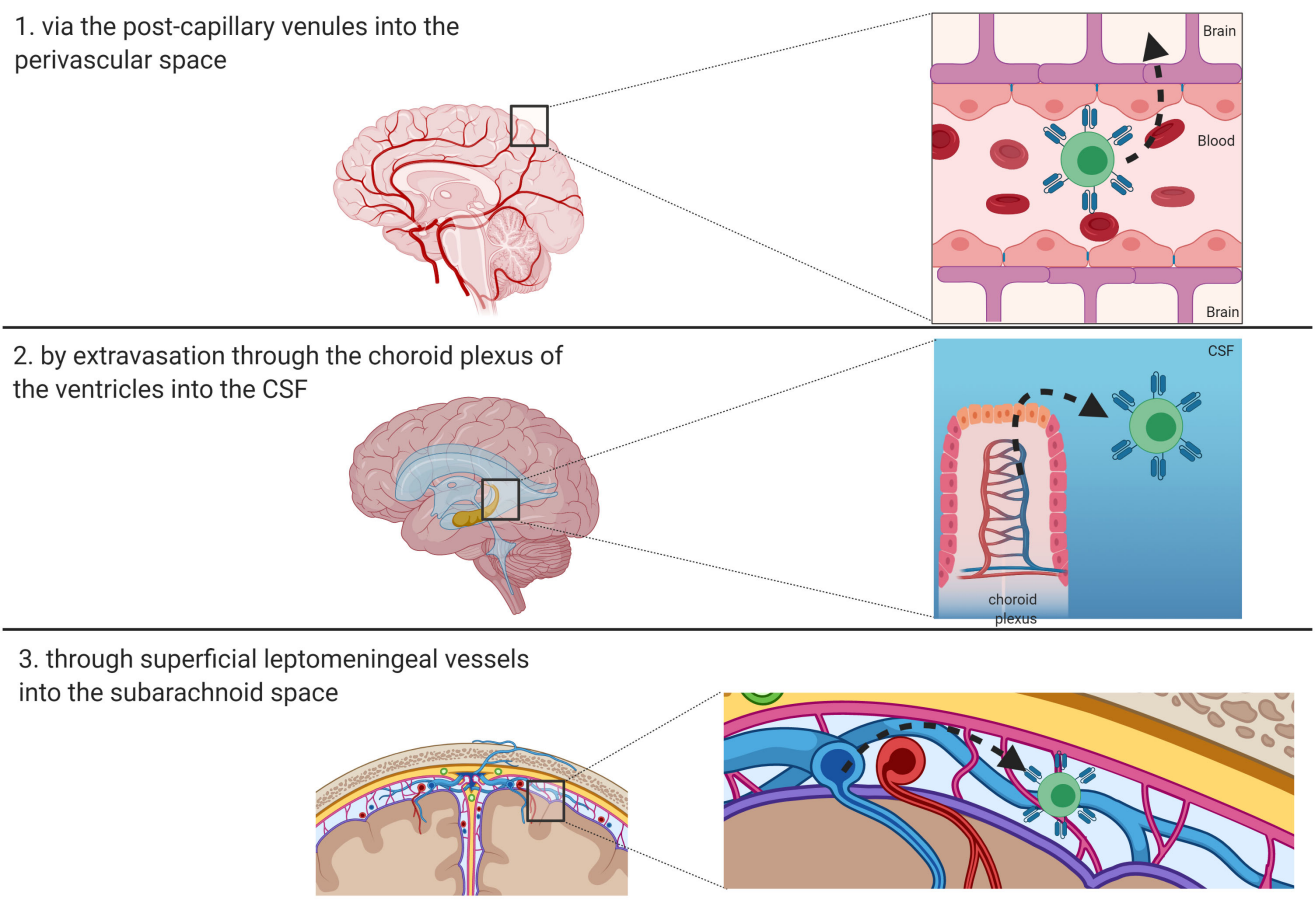
Route of entry of T-cells and CAR-T cells in the brain. Three main routes have been described and are depicted: 1) *via* the post-capillary venules into the perivascular space; 2) by extravasation through the choroid plexus of the ventricles into the CSF; or 3) through superficial leptomeningeal vessels into the subarachnoid space.

The BBB is a permeability barrier characterized by the connection, through tight junctions, of endothelial cells with the luminal and abluminal membranes lining the capillaries of the brain (40). The crucial role of BBB consists in protecting the brain from pathogens and finely tuning the brain homeostasis. Thanks to its low permeability, BBB prevents the entry of circulating ionic substances, large molecules and immune cells into the brain, as well as the passage of numerous therapeutic agents (41, 42). Therefore, T-cell penetration into the brain parenchyma involves complex mechanisms. Resting T cells do not cross the BBB, but traffic from meningeal blood vessels into the cerebrospinal fluid (CSF), where they can gain access to the brain parenchyma *via* the pia mater or choroid plexus. On the contrary, activated T cells seem to be able to traverse the capillary tight junctions of the BBB (43). The entry routes of lymphocytes and antigen presenting cells in the CNS are essentially three, namely: 1) *via* the post-capillary venules into the perivascular space; 2) by extravasation through the choroid plexus of the ventricles into the CSF; or 3) through superficial leptomeningeal vessels into the subarachnoid space (**Figure 2**) (44). The first step in the recruitment of T-cells is represented by the binding of integrins α4β1 and lymphocyte associated antigen-1 (LFA-1) expressed on activated T-cells to the adhesion molecules known as vascular cell adhesion molecule 1 (VCAM1) and intracellular cell adhesion molecule 1 (ICAM1) on endothelial cells, respectively (45). In the CNS, adhesion of T-cells to endothelial cells also involves a specific adhesion molecule, the activated leukocyte adhesion molecule (ALCAM), which binds CD6 on mature T-cells (46). The ligand-binding interactions lead to conformational changes in T-cells that promote their crossing through the endothelial lining into the perivascular space. Activated Tcells must ultimately traverse the glia limitans to enter the brain parenchyma. Subsequently, the penetration of T-cells into the brain parenchyma is regulated by matrix metalloproteases (MMPs) and other soluble factors, including tumor necrosis factor-α (TNFα), IL-12, TGFβ, and IL-6 (47, 48). The lymphocytes then exit the brain, to reach deep cervical lymph nodes, exploiting a recently discovered system of lymphatics in the meninges (49). These observations imply that T cells administered by systemic infusion can have access to tumors *via* the CSF and choroid plexus, overcoming the dogma that considers the brain as an immune sanctuary (49, 50). Indeed, this evidence is the basis for the encouraging results demonstrated in the neuro-immunology field using CAR-T cells, vaccines, and other forms of immunotherapy (50, 51). These approaches have the ability to target the tumor tissues, while sparing the normal surrounding brain parenchyma. Two advantages make CAR-T cells particularly attractive for brain tumors, as compared to other immunotherapy approaches: a) they do not require a functional systemic immune response, thanks to their intrinsic antitumor cytotoxicity; b) their efficacy does not correlate with the tumor mutational burden, depending upon the expression of the target antigen only to be activated (50, 52). Multiple trials using intravenously (i.v.) infused CD19-directed CAR-T cells have proven that CAR-T cells can cross the BBB, as they have been detected in the CSF *via* flow-cytometry and immunofluorescence post-treatment, suggesting that systemically administered CAR-T cell can be used in the neuro-oncology setting (12, 40).

Another system known to play a central role as guardian of the brain, beside BBB, is the glymphatic system. It is a distinct and privileged mechanism of CNS protection represented by a glial-dependent waste clearance pathway, replacing lymphatic vessels and draining away soluble waste proteins and metabolic products (53). Together with the BBB, glia limitans regulates lymphocyte entry into the brain (45). Specifically, microglia represents the essential myeloid resident population of the brain microenvironment, the equivalent of tissue macrophages in the CNS (54, 55). In the absence of any inflammatory stimuli, it origins from yolk sac myeloid progenitors and is crucial for brain development and for maintaining the sterility of CNS (56). When an inflammatory trigger occurs, circulating monocytes intervene to support microglia with mechanisms and functions not yet fully understood (57). Finally, microglia drains to the cervical lymph node *via* the glymphatic system running face to face to dural venous sinuses (58). To the best of our knowledge, no studies have analyzed microglia in the pediatric population, both in pathologic settings and in the healthy developing brain. Future research may exploit analysis of RNA sequencing to identify microglial subpopulation, potentially informing future strategies for microglia-targeted immunotherapy.

## 5 Strategies of delivery of CAR-T cells into the brain

Despite the documented ability of i.v. administered CAR-T cells to cross the BBB, targeted delivery of T cells at the level of the CNS is an attractive option to reduce systemic toxicity and increase CAR-T cell homing and concentration at tumor site.

Alternative strategies to directly deliver CAR-T cells to the CNS include: spinal intrathecal infusion, intraventricular infusion, intra-tumoral injection and disruption of the BBB by focal ultrasound.

Spinal intrathecal delivery is a well-known strategy in pediatric oncology (59): intermittent infusion of chemotherapy by lumbar puncture is a well-established therapy in pediatric oncology services and is well tolerated. Moreover, implantable chronic infusion devices are available, with different indications. A spinal subarachnoid catheter can be easily implanted and connected either to a subcutaneous reservoir for intermittent percutaneous injection or to a more sophisticated subcutaneous programmable infusion pump (60, 61).

An alternative option is represented by a subcutaneous implantable ventricular access device, a subcutaneous reservoir, connected to an intraventricular catheter that can be punctured under sterile technique (62, 63).

A further advantage of intrathecal infusion by implantable devices is the possibility to use them to obtain CSF samples easily. Potential drawbacks of intrathecal infusion include CNS infection, especially in the setting of subcutaneous implantable devices, and hemorrhage.

An additional strategy to circumvent the BBB consists in the intra-tumoral delivery of therapeutics. Recently, promising results have been reported using convection enhanced delivery (CED) systems, even in children (64, 65). CED is based on the concept of bulk flow, namely the tridimensional diffusion of molecules within the brain, pushed by a constant gentle pressure exerted by a microinjection pump. Injection catheters can be precisely positioned by stereotactic technique and connected to an implantable multi-channel infusion device (66). Coupling CED with computer simulation approaches has resulted in the possibility to accurately foresee the volumetric distribution of therapeutics within the brain, reaching a considerable target volume and a desirable volume shape when multiple injection catheters are positioned. Potential limitations include availability of the neurosurgical technical expertise to safely and precisely implant intra-tumoral catheters, possible clogging of infusion catheters with the therapeutic cell suspension and local complications, such as infection, CSF leak and bleeding. In addition, the effect of bulk flow on mass transfer might result in transient worsening of neurological signs and symptoms, limiting infusion speed. In fact, the accurate spatial distribution of injected therapy typical of CED might not be necessary for CAR-T cells.

Localized BBB modulation by focal ultrasound (FUS) has been recently proposed in the neuro-oncology field to increase the concentration of systemically infused chemotherapy at the tumor site (67, 68). FUS has the ability to induce an increase in the permeability of the BBB with the advantage of spatial selectivity within the CNS. This technique is based on the transcranial delivery of low frequency ultrasounds that determine oscillations of microbubbles, finally determining a temporary and reversible disruption of endothelial tight junctions, a phenomenon known as sonication (69). This concept can be applied both to magnetic resonance integrated systems, in which spatial targeting of the ultrasounds is guided by real time imaging, or to implantable subcutaneous docking systems, to which an external ultrasound source is connected whenever sonication is required. A limitation of FUS is the limited extension of sonication volumes that might require multiple treatment sessions and multiple subcutaneous docking systems implantation. Moreover, even if there is a growing number of preclinical experiences documenting its effectiveness in BBB disruption, there is little evidence to prove clinical benefit in neuro-oncology, especially in children (70).

Research using patient-derived orthotopic xenograft models showed that CAR T cell trafficking into the tumor site is not affected by the route of administration. However, loco-regional delivery provides greater antitumor activity and lower systemic pro-inflammatory cytokine production in these models (33). Studies comparing delivery routes in preclinical models of glioblastoma (GBM) have shown that local delivery outperforms systemic delivery of CAR-T cells. Intratumor administered IL13Rα2‐CAR-T cells resulted in long‐term survival in orthotopic GBM models, whereas IV delivery provided no significant benefit over control (71). Furthermore, when comparing locoregional delivery routes in a multifocal GBM model, intraventricular infusion exhibited improved targeting of multifocal disease (71).

The clinical experiences reported to date confirm these preclinical observations. Intrathecal (into the resected cavity of the tumor through a catheter device) and intraventricular administration of CAR-T cells were tested in a patient with multifocal GBM, proving well tolerated, without cytokine release syndrome (CRS) or severe neurotoxicity (52). Interestingly, the intraventricular delivery resulted into a better control of the disease, probably due to the improved trafficking to sites of multifocal disease by the delivery of cells into the cerebrospinal fluid. Similarly, the experience of Majzner et al. shows that the intracerebral route of administration of GD2-CAR T cells is feasible and associated with less systemic toxicity as compared to the intravenous administration (72).

The ideal application route might depend also from the molecular target. In particular, antigens with wide expression in normal tissues (i.e. HER2 and B7H3) may be associated with a significantly reduced toxicity after intracerebral administration (19). Overall, the evidence currently available from the few clinical experience favors the loco-regional administration. As mentioned above, pre-clinical and clinical experience demonstrated that loco-regional delivery of second‐generation IL13Rα2‐CAR T cells is safe and well‐tolerated, with no evidence of dose levels toxicities (19, 71). Systemic administration of HER2 CAR T cells showed an increased risk of severe pulmonary toxicities (73, 74). Conversely, the safety of the loco-regional administration of HER-2 CAR T cells has been demonstrated in the BrainChild-01 trial (75): multiple administration have shown to be feasible and tolerated and associated with peritumoral edema in MRI and increased pro-inflammatory cytokine production in CSF, consistent with CAR T-cell activity (76). GD2 infusion has been evaluated both loco-regionally and intravenously in several trials for brain tumors (see **Table 1**). As previously mentioned, Majzner et al. recently published a clinical experience with GD2 CAR-T cells in 4 cases of H3K27M-mutated diffuse midline gliomas. They found that intraventricular administration was associated with less systemic toxicity, high levels of cytokines and decreased immunosuppressive cell populations in CSF, compared with intravenous delivery (72). Concerning B7-H3, it is expressed on the surface of several normal tissues but the administration of B7H3-directed CAR T cells, either loco-regionally or systemically, was not associated with evidence of on-target/off-tumor toxicity in a preclinical, orthotopic mouse model (33, 77). Last, EGFRvIII antigen is not expressed in healthy tissue (78) and i.v. administration is not related to severe sides effects (73, 79, 80). Moreover, infiltrating anti-EGFRvIII CAR T cells were detected in the tumors of the patients after i.v. administration (80).

Several obstacles impair the homing of T cells in solid tumors and will also likely be faced in CNS tumors, regardless the limitation of the BBB. The dense extracellular matrix (ECM) of the tumor forms a physical barrier to the infiltration of CAR-T cells. Innovative strategies, based on engineering of T cells to express enzymes with lytic activity, including heparanase, have been proposed to overcome this obstacle (37, 38). Several groups have also explored the possibility of disrupting physical barriers in solid tumors developing CAR-T cells able to recognize antigens expressed on stromal cells, such as the fibroblast activation protein (FAP) (39, 81). Another strategy to improve T-cell infiltration relies on the deep characterization of the chemokine pattern associated with the different tumors and the generation of CAR-T cells expressing the relative chemokine receptors (CCRs) to enhance the tumor homing (81–83). Lastly, Adachi and colleagues developed a CAR-T cell construct able to produce IL-7 and CCL19, both implicated in the recruitment of T cells from the bloodstream in the T-cell zone of lymphoid organs. These modified CAR-T cells resulted in a higher anti-tumor activity and increased tumor infiltration (84).

Finally, the application route might depend from the cell product. In the era of adoptive cell immunotherapy several cell products are being considered. Even if autologous CAR-T cells are the only cell product currently tested in brain tumors, other cell products are emerging in the pre-clinical field and for other clinical applications (i.e. CD19-positive hematological malignancies). In particular, irradiated CAR-NK cells derived from a cell line have been explored *in vitro* and validated *in vivo* against CD19 malignant cells (85). Despite the known low risk of toxicity compared with T cells, CAR-NK cells have some important limitations such as: short life-time, reduced cytotoxicity *in vivo* (85). We can hypothesize that these intrinsic characteristics would favor their use *via* loco-regional delivery, although some clinical evidence on the use of CAR-NK cells have shown promising results (86). An interesting advantage of NK cells relies on the possibility of deriving the product from allogeneic donors, as an “off the shelf product” (87, 88).

## 6 Pediatric brain tumor-specific toxicities: Beyond common neurotoxicity and possible strategies of monitoring and treatment

The potential for CAR-T cell therapies to induce life-threatening neurotoxicity has been long-appreciated by researchers in the field (89, 90), but CNS-directed CAR-T cell therapies could induce additional neurologic damage as collateral effects of inflammation arising in the setting of tumor-directed therapy. By targeting tumors that have infiltrated critical brain structures, adoptive T-cell therapies induce inflammation, leading to tumoral and peritumoral edema. This localized edema can alone induce neurologic symptoms. If edema leads to mass effect and compromises blood or cerebrospinal fluid circulation, ischemia or life-threatening increases in intracranial pressure can rapidly develop.

Recently, neurotoxicity correlated to the use of immunotherapies has been renamed as immune effector cell-associated neurotoxicity syndrome (ICANS), and it is the second most frequent event after CRS. Described in about 37% of the patients treated with CD19-CAR-T cells for B-cell malignancies (91), it is characterized by a wide spectrum of neurological impairments, ranging from mild to severe manifestations, including headache, dizziness, irritability, memory loss, aphasia, tremor until seizures and global encephalopathy (6, 91–94). In patients with brain tumors, neurotoxicity can be a more challenging side effect than in patients with leukemia due to the peculiar primary site of the disease in CNS. Therefore, treating CNS tumors with CAR-T cells imposes to carefully consider these specific toxicity scenarios.

Infiltration of tumor tissue by immune cells is an expected and desired effect of the treatment. In fact, transient tumor volume increase, a phenomenon known as pseudo-progression, has been largely described with immunotherapies (95). However, this mass effect has to be taken into account when selecting potential candidates. The intracranial volume reserve (i.e. the ability to tolerate an expanding mass inside the skull) is limited, especially in patients with CNS malignancies, who already experience an increase of the intracranial volume related to the mass itself and the surrounding edema. Unfortunately, there is little data available in the literature to aid in defining with sufficient precision the intracranial volume reserve in a specific patient. Post-mortem studies suggest it accounts for about 5% on the total cranial volume in young adults, thus being equivalent to about 65-75 ml, with smaller volumes found in females (96). Volume reserve might be significantly lower in children and patients with pre-existing intracranial mass lesions.

Most of the compensatory volume following the presence of a growing intracranial mass is secondary to CSF redistribution. The compensation ability of intracranial pressure depends not only on volume itself, but also on volume change/time rate: a slow growing large mass is clinically much better tolerated than a fast-growing smaller lesion. Tumor location deserves a careful evaluation when considering CAR-T cell treatment in CNS tumors. Intracranial mass effect increase will potentially be more intimidating for deep seated lesions (midline thalamic tumors, brainstem and posterior fossa location) where the risk of trans-falcine and trans-tentorial pressure cones determining brain herniation and hydrocephalus is higher. The ideal, opposite scenario would be a leptomeningeal disseminated disease, with a much lower potential for intracranial hypertension development. Although the risks of CNS inflammation also involve the adults, it represents a peculiar issue in the pediatric setting because of the different intracranial volume reserve. In particular, in younger children, the unclosed cranial suture allows for a larger reserve which, on one side, offers a greater adaptation to the increase of intracranial pressure, but, on the other side, is responsible for the more insidious symptoms associated to the occurrence of intracranial hypertension. These elements make the identification of a more sophisticated and early monitoring system particularly relevant in the pediatric setting.

Despite limited possibilities for determining non-invasively the CSF compensatory volume, there is evidence in the literature about the predictive value of invasive intracranial pressure waves analysis. Specific wave morphology might suggest a reduction of the buffer volume, prompting appropriate therapeutic countermeasures (97).

Several strategies can be hypothesized in order to manage this possibly severe side effect of the treatment. A ventricular access device might be inserted before infusion of CAR-T cells and used both to directly assess intracranial pressure waves and to remove determined CSF volumes, if appropriate to improve intracranial pressure management. Moreover, whenever a high risk of decompensation secondary to CAR-T cell treatment is suspected based on significant mass effect of a CNS lesion, preemptive surgical debulking might be an option, in selected cases. Lastly, in case of need of aggressive treatment of intracranial pressure, surgical decompressive procedures might be considered. However, as well-known from neurotrauma and malignant stroke literature, functional outcomes might be unacceptable, raising ethical issues for invasive maneuvers in fragile patients with an overall dismal prognosis (98–101).

Other strategies can be considered in order to reduce the risk of severe, life-threatening increase of intracranial pressure secondary to pseudo-progression. The sequential administration of low doses of CAR-T cells might result in a lower tumor infarction with CAR-T cells and a relatively slow and progressive tumor lysis, reducing the risk of a sudden intracranial hypertension. In addition, the use of different cell platform, with a lower persistence over time and a reduced inflammatory profile upon activation, such as NK cells, could reduce the risk associated with the infusion of these cells. Similarly, the use of transiently expressed CAR-T cells, generated by RNA electroporation, might represent a valuable risk mitigation strategy. Lastly, the introduction of a suicide gene (i.e. inducible caspase 9 or Herpes Simplex Virus-1 Thymidine Kinase) (102, 103), capable to rapidly induce the apoptosis of CAR-T cells and, thus, to mitigate the inflammation and the pseudo-progression, represents an attractive option for increasing the safety profile of the approach.

## 7 Clinical translation in future

As already mentioned, the hurdles to be overcome in order to develop CAR T cell approaches for brain tumors are many and insidious, including, but not limited to: i) tumor heterogeneity, ii) limited persistence and trafficking, iii) adaptive immune resistance, iv) immunosuppressive microenvironment. Many efforts still need to be made to obtain concrete results for future neuro-oncology applications.

Recent molecular characterization of gliomas and other high-grade tumors of the central nervous system have led to a better understanding of the heterogeneity of these diseases and the complex mechanism of interaction between tumor cells and microenvironment (104–106). Constructs targeting multiple antigens simultaneously can be more effective but certainly more challenging, being associated with higher risk of off-tumor toxicity (36, 107). The validation and application of these targets in the clinical setting is a daunting challenge that requires the joint work of multidisciplinary clinical teams and laboratory researchers to achieve the best results.

To reduce CAR T resistance in brain tissue, various alternative approaches could be considered, for example: CAR T cells designed to co-express dominant-negative TGFβRII, which lock up TGFβ signaling within the engineered T cells (108); EBV specific cytotoxic T lymphocytes transduced with TGFβRII with the aim to produce cytokines and maintain cytolytic response (109); chimera CAR T cells expressing a fusion molecule, including IL-4 ectodomain and IL-7 endodomain, promoting T-cell proliferation and maintaining antitumor effect (110).

To prolong the persistence, CAR T cells can be armed to deliver cytokines, with the goal of supporting T persistence and remodeling TME toward pro-inflammatory environment (111–116).

Moreover, combination therapies to improve CAR T cell function have been investigated recently. Targeting immune inhibitory antigens such as PD-1, PD-L1 and CTLA-4 with either nivolumab, pembrolizumab or ipilimumab have been tested in clinical studies focused on GBM, showing promising results in terms of both safety/feasibility and anti-tumor activity (117–121). Oncolytic viruses genetically modified to target a suppressive TME (122, 123) and agonist antibodies specific for the 4-1BB costimulatory receptor can also potentially amplify CAR T cell efficacy by counteracting the immune-suppressive microenvironment (124). In addition, the hypoxia transcription factor HIF-1a subdomain can be incorporated in a CAR construct to reduce on-target off-tumor toxicity, ensuring CAR T cells activation only under the hypoxic conditions characteristic of the TME (125).

Lastly, although limited data are available in brain tumors, strategies to improve T cell trafficking and infiltration by engineering the cells to express tumor homing receptors are promising, as demonstrated in other types of solid tumors. In particular, CD70-specific CAR T cells expressing CXCR1 and CXCR2 have demonstrated improved T cell trafficking and efficacy, in preclinical models of GBM (126). Additional promising pathways that can be exploited to enhance CAR T cell accumulation at tumor site are represented by endothelial adhesion molecules and vascular cytokines (114, 127).

## 8 Conclusions

CAR-T cells are emerging as a promising treatment for pediatric brain tumors, raising specific challenges for the management of these delicate patients. Published evidences are still extremely scarce, but they offer the opportunity to start a careful, although still largely speculative, consideration of the peculiar issues associated to the administration of this treatment in a peculiar location such as the brain. Although there is no unequivocal evidence on the best route of delivery, systemically administered CAR-T cells can be used in the neuro-oncology setting, thanks to the increasing evidence of T-cell ability to infiltrate CNS. However, intrathecal and/or intra-tumoral delivery can be easily managed, due to implantable devices, and early evidence suggests that it might represent a more efficient and less toxic option for these diseases.

Concerns related to the peculiar toxicities associated with the treatment of these tumors need to be carefully evaluated and properly handled thanks to a multi-disciplinary approach. Advances in the monitoring and managing of intracranial pressure, a scrupulous selection of the patients and the optimization of the CAR products, by the incorporation of a suicide gene, as well as by the development of products with a limited half-life, mitigate these risks, making the approach more manageable.

Progress in immunotherapy is raising optimism in the neuro-oncology field. The integration of advanced preclinical studies in current clinical practice may truly bring the promise of CAR-T cell therapy to the conventional armamentarium of the neuro-oncologist.

## Author contributions

AM and FL conceptualized the work. GB and FB wrote the manuscript. CP, PM, ACac contributed to data collection and figures realization. ACar, CQ, BA, FL and AM contributed to the finishing of the work and revised it critically for important intellectual content. All authors contributed to the article and approved the submitted version.
